# A data calibration method for micro air quality detectors based on a LASSO regression and NARX neural network combined model

**DOI:** 10.1038/s41598-021-00804-7

**Published:** 2021-10-27

**Authors:** Bing Liu, Yueqiang Jin, Dezhi Xu, Yishu Wang, Chaoyang Li

**Affiliations:** 1 Public Foundational Courses Department, Nanjing Vocational University of Industry Technology, Nanjing, 210023 China; 2 Organization Department, Nanjing Vocational University of Industry Technology, Nanjing, 210023 China; 3 College of Management, Henan University of Technology, Zhengzhou, 450001 China

**Keywords:** Atmospheric science, Climate sciences, Environmental sciences

## Abstract

Studies have shown that there is a certain correlation between air pollution and various human diseases, especially lung diseases, so it is very meaningful to monitor the concentration of pollutants in the air. Compared with the national air quality monitoring station (national control point), the micro air quality detector has the advantage that it can monitor the concentration of pollutants in real time and grid, but its measurement accuracy needs to be improved. This paper proposes a model combining the least absolute selection and shrinkage operator (LASSO) regression and nonlinear autoregressive models with exogenous inputs (NARX) to calibrate the data measured by the micro air quality detector. Before establishing the LASSO-NARX model, correlation analysis is used to test whether the correlation between the concentration of air pollutants and its influencing factors is significant, and to find out the main factors that affect the concentration of pollutants. Due to the multicollinearity between various influencing factors, LASSO regression is used to further screen the influencing factors and give the quantitative relationship between the pollutant concentration and various influencing factors. In order to improve the prediction accuracy of pollutant concentration, the predicted value of each pollutant concentration in the LASSO regression model and the measurement data of the micro air quality detector are used as input variables, and the LASSO-NARX model is constructed using the NARX neural network. Several indicators such as goodness of fit, root mean square error, mean absolute error and relative mean absolute percent error are used to compare various air quality models. The results show that the prediction results of the LASSO-NARX model are not only better than the LASSO model alone and the NARX model alone, but also better than the commonly used multilayer perceptron and radial basis function neural network. Using this model to calibrate the measurement data of the micro air quality detector can increase the accuracy by 61.3–91.7%.

## Introduction

With the development of science and technology, the progress of industry and the rapid increase of the global population, the environment that people depend on has been greatly destroyed. Many areas have experienced environmental problems such as acid rain, species extinction, and land desertification. Environmental issues have become one of the common concerns of all countries in the world today, and they are also a major challenge facing humanity in the twenty-first century. Air pollution is an especially concerning environmental issue, which can easily lead to respiratory diseases such as acute and chronic bronchitis, asthma, pneumonia, and even lung cancer^1–3^. According to estimates by the World Health Organization, 7 million people die each year from diseases caused by air pollution^4,5^.

The pollutants in the air are mainly inhalable particles, SO_2_, NO_2_ and other substances. The commonly used index to measure the quality of air is AQI, which is the Air Quality Index. The larger the AQI value, the more serious the air pollution, and the greater the harm to human health. AQI (GB3095-2012) is calculated based on six air pollutants: PM_2.5_, PM_10_, CO, NO_2_, SO_2_ and O_3_ (“two dusts and four gases"). As air quality is getting more and more attention, it is particularly important to monitor air quality.

In order to monitor the air, several national air quality monitoring stations (national control points) are generally set up in a key environmental protection city. Multi-parameter automatic monitoring equipment is installed in the air quality monitoring station for continuous automatic monitoring, and the monitoring results are stored in real time and analyzed to obtain relevant data. The construction and maintenance costs of national control points are relatively high, so the number of national control points is very small, which makes it difficult to conduct comprehensive monitoring of an area. In addition, although the national control point data is relatively accurate, it is often not released in real time, so it is difficult to realize real-time monitoring of air quality. In order to overcome the deficiencies of grid monitoring and real-time monitoring of pollutant concentration at national control points, some companies have developed miniature air quality detectors, which have the advantages of low cost, convenient installation, and convenient data reading. It can be deployed more intensively than national control points, and can also be evenly grid-arranged in key areas, which has achieved the purpose of grid-based monitoring^6–8^. However, since the electrochemical sensor used in the micro air quality detector is susceptible to external influences, the range drift and zero point drift will occur after a period of use, and the data measured by the self-built point will have a certain error. How to use the national control point data to calibrate the self-built point data is a problem worthy of study.

The commonly used pollutant concentration prediction models are mainly divided into two categories. The first type is the atmospheric chemistry transmission model, which uses the theory of the atmospheric system to simulate the physical and chemical processes of pollutants in a specific area, and uses the generated pollutant grid data to predict air quality^9,10^. The mechanism of the atmospheric chemistry transmission model is complex, and is limited by the accuracy of the ground emission inventory, and its pollutant forecast effect is not very good.

Another commonly used pollutant concentration prediction model is a statistical model based on machine learning algorithms. The multiple linear regression model is a relatively classic statistical model, which can give a quantitative relationship between the concentration of pollutants and various influencing factors. The regression equation established based on these quantitative relationships can effectively predict the concentration of pollutants. If necessary, the concentration of pollutants can be effectively controlled or dealt with according to these factors. Because the multiple linear regression model has good interpretability, the construction of multiple linear regression equation is still a common air quality prediction modeling idea^11,12^. Lei et al. used meteorological and air quality data from 2013 to 2017 for five years to establish a statistical model based on linear multiple regression (MR) and classification regression tree (CART) analysis. The model successfully predicted the concentrations of NO_2_, PM_10_, PM_2.5_ and O_3_ in Macau on the second day^13^. For the multicollinearity problem that may exist in the construction of multiple regression model, least absolute selection and shrinkage operator (LASSO) regression is one of the methods often used to solve it. Sethi et al. proposed an adaptive LASSO regression method based on correlation, successfully identified the important factors affecting the air quality index, and completed the forecast of air quality in Delhi^14^. It is difficult for multiple linear regression models to detect the complex and potentially non-linear relationship between predictor variables and response variables, so machine learning algorithms such as artificial neural networks^15–18^, support vector machines^19–22^, random forest^23–26^ and extreme gradient boosting^27–29^ have become the mainstream of pollutant concentration prediction. The nonlinear autoregressive models with exogenous inputs (NARX) increases the delay and feedback mechanism, so it enhances the ability to remember historical data. In recent years, it is often used for air quality prediction. Moursi et al. used the PM_2.5_ concentration, cumulative wind speed and cumulative rainfall hours in the past 24 h as independent variables, and successfully predicted the PM_2.5_ concentration in the next hour using the NARX model^30^. Mohebbi et al. successfully simulated the carbon monoxide concentration in Shiraz using the NARX neural network model without traffic data. The results show that the dynamic neural network is better than the static neural network in the prediction accuracy of CO concentration in this area^31^.

**Figure 1:**
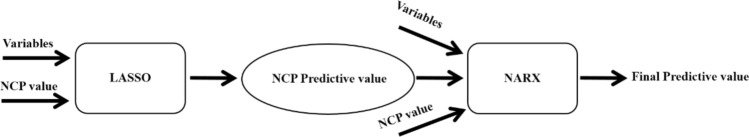
The flux diagram of the regression process, where NCP represents the concentration of pollutants measured at the national control point.

There are many factors that affect the concentration of pollutants, and each factor has a mutual influence. If all factors are directly introduced into the multiple linear regression model, multicollinearity may occur. LASSO regression can improve the multicollinearity of the model and retain the interpretability of the multiple linear regression model. The advantage of NARX neural network over LASSO model is that it can find out the nonlinear relationship between pollutant concentration and various influencing factors. Therefore, the NARX neural network has higher prediction accuracy than the LASSO model. Combining the LASSO regression model and NARX neural network can not only retain the advantages of the two models, but also make full use of the data measured by the micro air quality detector. This combined model is called the LASSO-NARX model in this paper. The empirical results show that the LASSO-NARX model can not only improve the interpretability of the NARX model, but also improve the prediction accuracy of the LASSO model. Figure 1 shows the construction process of the LASSO-NARX model.

## Material and methods

### Data source and preprocessing

The appearance of the micro air quality detector makes it possible to monitor the concentration of pollutants in real time, but the accuracy of its measurement needs to be improved. The two sets of data are collected in this paper to build the data calibration model of the micro air quality detector. The first set of data is measured by a national monitoring station in Nanjing, which provides the concentration of two dusts and four gases from November 14, 2018 to June 11, 2019. It has a total of 4200 pieces of data, and the interval of each group of data is mostly 1 h. The second set of data is measured by a self-built point equipped with a micro air quality detector. It contains 234,717 pieces of data whose time interval does not exceed 5 min. The location of the self-built point is within 10 m from the national control point. It not only measures the concentration of the two dust and four gases in the same period, but also provides five meteorological parameters of wind speed, pressure, precipitation, temperature and humidity.

Preprocessing of data is a prerequisite for building statistical models. The first step is to delete duplicate data and obviously abnormal data (greater than three times the average value of the left and right neighbors) in the data. In the second step, the self-built point data is averaged on an hourly basis, and the averaged self-built point data is used to correspond to the national control point data, and the data that cannot be corresponding is deleted. The summary table of self-built point data and national control point data after preprocessing is shown in Table 1.

**Table 1:** Descriptive statistics of pollutant concentrations and meteorological parameters measured by national control points and self-built points after pretreatment.

Input variable	Ranges	Mean	Standard deviation	Skewness	Kurtosis
PM_2.5_ (μg/m^3^)	1–216.883	64.127	37.328	0.988	0.701
PM_10_ (μg/m^3^)	2–443.25	102.391	65.267	1.476	2.862
CO (μg/m^3^)	0.05–3.895	0.863	0.452	1.463	3.136
NO_2_ (μg/m^3^)	0.947–157.136	45.209	28.403	0.653	− 0.259
SO_2_ (μg/m^3^)	1–651.3	19.397	18.723	12.781	342.11
O_3_ (μg/m^3^)	0.579–259	61.586	40.941	1.091	2.035
Wind speed (m/s)	0.133–2.387	0.7	0.346	0.862	0.748
Pressure (Pa)	996.871–1039.8	1018.8	8.889	− 0.093	− 0.599
Precipitation ( mm/m^2^)	0–312.1	132.084	87.004	0.245	− 0.728
Temperature (^o^C)	− 3.882–37.944	11.882	8.603	0.625	− 0.399
Humidity (rh%)	10.667–100	68.903	21.931	− 0.487	− 0.756

### Data exploratory analysis

Due to the influence of internal factors and external factors, there are certain errors in the data measured by the micro air quality detector. This article draws a time series chart to show the difference between self-built point and national control point^20,32^. The discussion method of the two dusts and four gases is similar. We randomly select O_3_ for analysis.

**Figure 2:**
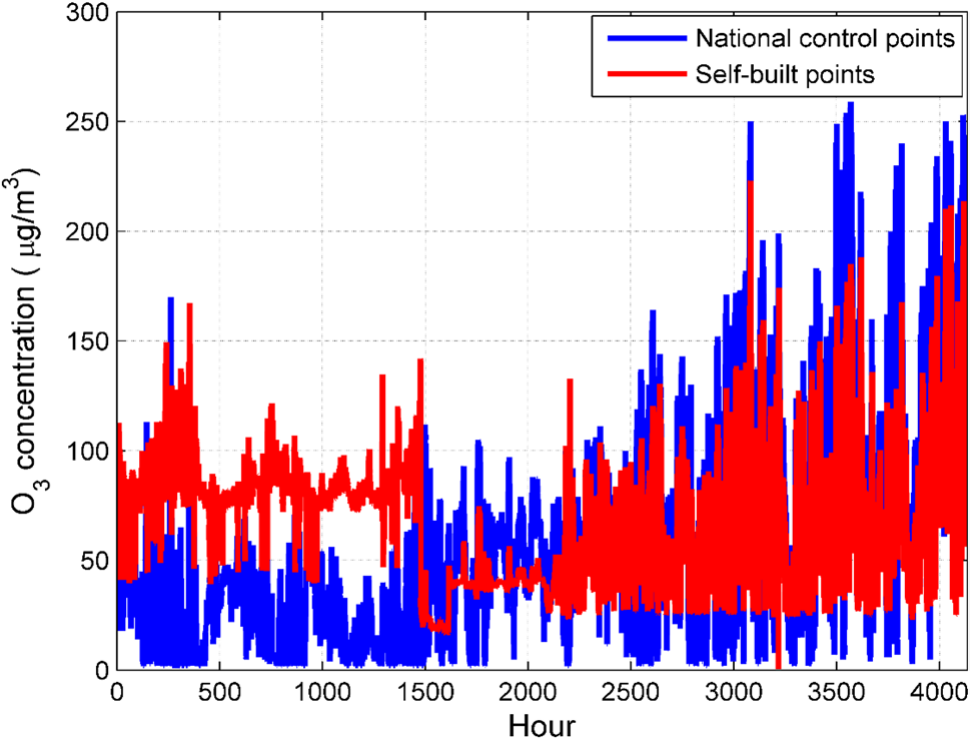
Comparison of hourly average O_3_ concentration data between national control points and self-built points. Figures are generated using Matlab (Version R2016a, https://www.mat- hworks.com/) [Software].

It can be seen from Fig. 2 that the change trend of O_3_ concentration at the self-built point is roughly the same as that at the national control point. However, there is a certain difference between the O_3_ concentration of the self-built point and the national control point. In the first 1500 h, the O_3_ concentration of self-built point was generally higher than that of national control points. After 1500 h, the fluctuation degree of O_3_ concentration at the national control point is generally greater than the fluctuation degree of the O_3_ concentration at the self-built point.

**Figure 3:**
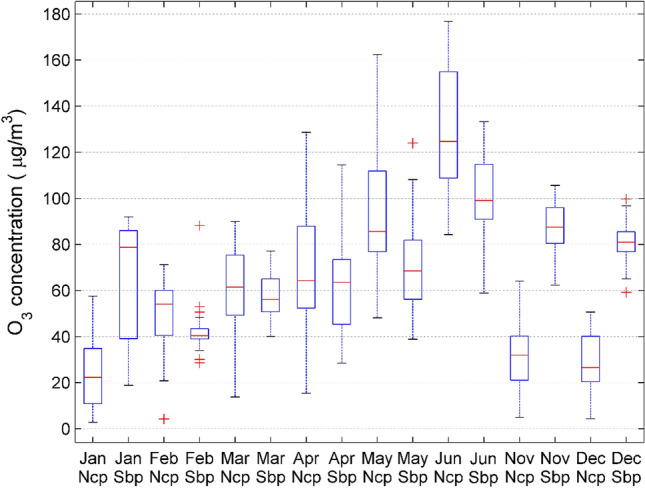
Compare the O_3_ concentration of national control points (Ncp) and self-built points (Sbp) on a monthly basis. Note that there is no data from July to October.

Since there are certain differences in meteorological parameters in each month, in order to reflect the influence of meteorological parameters on the concentration of pollutants, we have drawn a box plot^33^ as shown in Fig. 3. It can be seen that the difference in O_3_ concentration between self-built point and national control point is different every month. In November, December, January and February, the O_3_ concentration difference between the self-built point and the nationally controlled point is large. The reason is that the low temperature and low humidity during this period affect the accuracy of the electrochemical sensor. It can be seen that meteorological parameters are also factors that affect the concentration of pollutants.

### Correlation analysis

The key to air quality prediction is the prediction of the concentration of pollutants such as two dusts and four gases. Predicting the concentration of pollutants must find out the main factors that affect it^10^. Because the factors that affect the concentration of pollutants in the air are more complex, and the factors themselves also affect each other, quantitative indicators are needed to describe them. Pearson correlation coefficient (Eq. (1)) is a statistical indicator used to reflect the degree of correlation between variables^13,29^.

Table 2 shows the correlation between the concentration of six types of pollutants and meteorological parameters. It can be seen that at a significant level of 0.05, all variables have a significant correlation with each other except for the NO_2_ concentration and temperature. The absolute value of the correlation coefficient between many of these variables exceeds 0.8, indicating that they are highly correlated.

**Table 2:** Pearson linear correlation coefficients between six types of air pollutant concentrations and meteorological parameters (Band * indicates significant correlation at a significant level of 0.05).

Variable	PM_2.5_	PM_10_	CO	NO_2_	SO_2_	O_3_	Wind speed	Pressure	Precipitation	Temperature	Humidity
PM_2.5_	1.00	0.89*	0.66*	0.26*	0.29*	− 0.26*	− 0.23*	0.89*	− 0.70*	− 0.16*	0.18*
PM_10_		1.00	0.63*	0.34*	0.35*	− 0.19*	− 0.18*	0.38*	− 0.10*	− 0.03*	− 0.09*
CO			1.00	0.30*	0.31*	− 0.27*	− 0.31*	− 0.07*	0.08*	− 0.05*	0.22*
NO_2_				1.00	− 0.34*	− 0.26*	− 0.36*	− 0.10*	− 0.14*	− 0.02	− 0.11*
SO_2_					1.00	− 0.28*	− 0.19*	0.19*	0.27*	− 0.10*	0.11*
O_3_						1.00	0.39*	− 0.45*	− 0.12*	0.68*	− 0.62*
Wind speed							1.00	0.09*	0.06*	0.07*	− 0.32*
Pressure								1.00	0.23*	− 0.85*	0.15*
Precipitation									1.00	− 0.14*	0.86*
Temperature										1.00	− 0.49*
Humidity											1.00

1 $$r=\frac{\sum_{i=1}^{n}({x}_{i}-\overline{x})({y}_{i}-\overline{y})}{\sqrt{{\sum_{i=1}^{n}({x}_{i}-\overline{x})}^{2}}\cdot \sqrt{{\sum_{i=1}^{n}({y}_{i}-\overline{y})}^{2}}}$$

## Establishment of sensor calibration model

### Introduction to basic principles

Least absolute selection and shrinkage operator was first proposed by Tibshirani in 1996. This method is a compression estimation. It constructs a penalty function to obtain a more refined model, so that it can compress some coefficients, and at the same time set some coefficients to zero, to achieve the effect of subset shrinkage^29,34^.

In a general regression model, the observed values of each data are generally considered to be independent of each other. Because there are many variables in the model, their dimensions are often different. In order to eliminate the interference of dimensions, all independent variables ${X}_{i}=({x}_{i1},{x}_{i2},\cdots ,{x}_{im})$ need to be standardized via a linear transformation. The standardized ${X}_{i}^{*}=({x}_{i1}^{*},{x}_{i2}^{*},\cdots ,{x}_{im}^{*})$ mean is 0, and the variance is 1. Equation (2) is the LASSO estimate of the regression model, where the second term is the L1 penalty, $k$ is a nonnegative regularization parameter. When $\mathrm{k}=0$, LASSO regression is ordinary least squares regression. With the increase of $\mathrm{k}$, the LASSO can compress the coefficients of unimportant variables to 0, thus realizing variable selection. The larger the value of k, the more parameters are compressed to 0, and the smaller the model complexity, which solves the problem of poor model interpretability^14,35,36^.

2 $$\widehat{\beta }(\mathrm{LASSO})=\mathrm{arg}\underset{\beta }{\mathrm{min}}{\Vert y-\sum_{j=1}^{p}{x}_{j}{\beta }_{j}\Vert }^{2}+k\sum_{j=1}^{p}\left|{\beta }_{j}\right|$$

A typical NARX neural network is mainly composed of input layer, hidden layer, output layer and input and output delay. NARX neural network model is a kind of nonlinear discrete system, which can be represented by a nonlinear difference equation (Eq. (3)), where $y$ represents the output variable; $x$ represents the external input variable; $d$ represents the delay step. Different delay steps can be set for output variables and input variables to control the time step of continuous prediction.

Equation (4) is the calculation formula for the output of each layer, where ${x}_{i}$ represents the input of each layer of neurons, that is, the output of the previous layer of neurons; ${a}_{i,j}$ represents the weight between layers;${b}_{j}$ represents the threshold of the layer; $f$ represents the activation function. The activation function of the hidden layer of the NARX neural network uses the hyperbolic tangent function (Eq. (5)), and the output layer uses the linear function (Eq. (6)).

3 $$y\left(t\right)=f(x\left(t-1\right),x\left(t-2\right),\cdots ,x\left(t-d\right),y\left(t-1\right),y\left(t-2\right),\cdots ,y\left(t-d\right))$$

4 $${H}_{j}=f(\sum_{i=1}^{n}{a}_{i,j}{x}_{i}-{b}_{j})$$

5 $$\mathrm{tanh}\left(x\right)=\frac{{e}^{x}-{e}^{-x}}{{e}^{x}+{e}^{-x}}$$

6 $$\mathrm{linear}\left(x\right)=x$$

### LASSO regression model construction

From the correlation analysis, we can see that there is a strong correlation between the concentration of various pollutants, and between the pollutants and meteorological parameters. In this paper, the pollutant concentration at the national control point is used as the dependent variable, and the pollutant concentration and meteorological parameters measured at the self-built point are used as independent variables to establish a multiple linear regression model. An important requirement of multiple linear regression models is that the independent variables are independent of each other. The variance inflation factor is often used to determine whether the variables of a model are independent of each other. Let the standardized independent variable be ${X}^{*}$, then X*′X*=(*r*_*ij*_) is the correlation matrix of the independent variable. The main diagonal element of the (X*′X*)^−1^ is defined as the variance inflation factor of the independent variable. Through the multicollinearity diagnosis of the model, we can see that the maximum variance inflation factor of the multiple linear regression model is 26.631, which is greater than 10. Therefore, the multiple linear regression model has serious multicollinearity. Multicollinearity will make the air quality prediction model very unstable and cause over-fitting problems.

Commonly used methods to solve multicollinearity in practical problems are: (i) Selecting the independent variables, and the representative methods include forward regression, backward regression and stepwise regression. (ii) Perform dimensionality reduction processing on independent variables. Representative methods include principal component regression and partial least squares regression. (iii) Biased estimation of regression coefficients, representative methods include ridge regression and LASSO regression. This study uses LASSO regression to solve the problem of multicollinearity. Compared with ridge regression, LASSO regression can select variables and eliminate some variables that have no significant influence on the dependent variable. Compared with stepwise regression, LASSO regression can retain those variables that are between significant and non-significant effects on the dependent variable, so the estimation deviation is not too large.

In the process of establishing the LASSO regression model with the help of SPSSAU (https://spssau.com/) software, in order to facilitate comparison with other models, we randomly selected 85% of the data to build the model, and the remaining 15% of the data for model verification. The analysis of LASSO regression using SPSSAU software is divided into two steps: (i) Find the best k value based on the trajectory graph. The selection principle of k value is the minimum k value when the standardized regression coefficient of each independent variable becomes stable. The smaller the k value, the smaller the deviation, when the k value is 0, it is an ordinary linear OLS regression. (ii) Manually input k value for regression modeling. For the k value, generally the smaller the better, and it is generally recommended to be less than 1.After determining the k value, we can manually enter the k value to get the LASSO regression model estimate.

**Figure 4:**
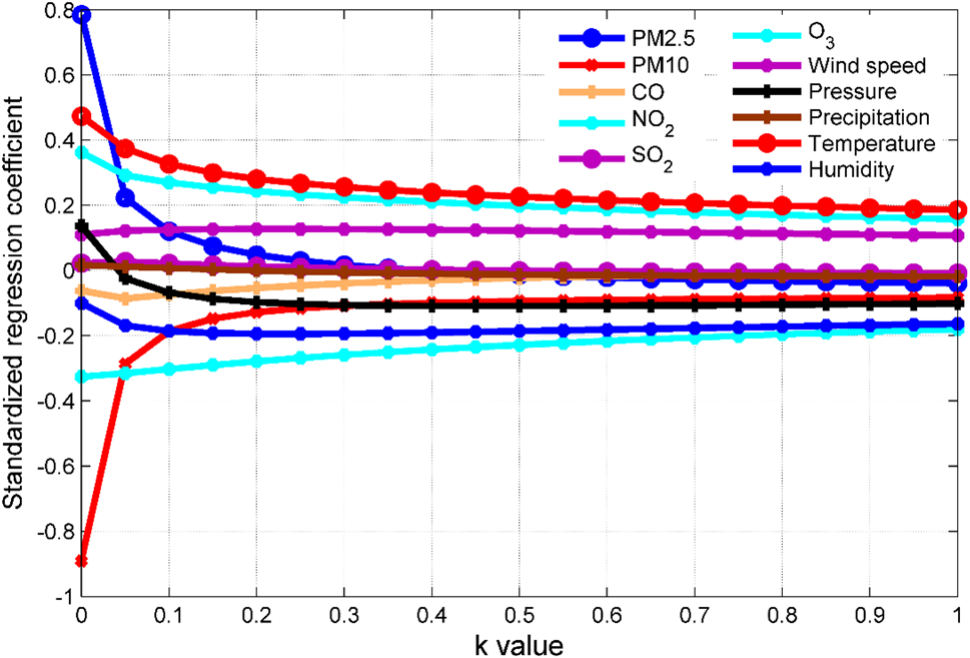
The trace diagram of all input variables, where the dependent variable is the O_3_ concentration measured by the national control point.

For the LASSO regression model of O_3_ concentration prediction, it can be seen from Fig. 4 that when k = 0.05, the standardized regression coefficients of each independent variable tend to be stable, so this paper takes k = 0.05 to establish the LASSO regression model. In the model, PM_2.5_ concentration, CO concentration, SO_2_ concentration, pressure and precipitation have no effect on O_3_ concentration, so they are excluded from the model.

7 $$F=\frac{SSR/s}{SSE/(n-s-1)}$$

8 $$SSR=\sum_{i=1}^{n}{({w}_{i}-\overline{y })}^{2}$$

9 $$SSE=\sum_{i=1}^{n}{({y}_{i}-{w}_{i})}^{2}$$

10 $${R}^{2}=1-\frac{\sum_{t=1}^{n}{({y}_{t}-{w}_{t})}^{2}}{\sum_{t=1}^{n}{({y}_{t}-\overline{y })}^{2}}$$

After the LASSO model is established, the model needs to be tested. Equations (7)–(9) are the definitions of F value in F test, where s represents the number of introduced model variables, n represents the total number of samples, ${y}_{i}$ represents the true value, ${w}_{i}$ represents the model fitted value, and $\overline{y }$ represents the average value of the true value. P value is more convenient for model verification. The P value is the probability of a sample observation or extreme result when the null hypothesis is true (the null hypothesis here is that the variables introduced into the model have no significant effect on the dependent variable as a whole). Equation (10) is the formula of the model’s goodness of fit, which reflects the degree of fit of the regression line to the observed value. The F value in the model test is 1123.756, and the corresponding p value is less than 0.01, indicating that at the significance level of 0.01, the overall variables introduced into the model have a significant impact on the pollutant concentration. The coefficient of determination of the LASSO model is 0.750, indicating that 75% of the change in O_3_ concentration can be explained by the change in the independent variables introduced into the model. The results of the remaining pollutants LASSO regression model are shown in Table 3.

**Table 3:** LASSO regression model of six types of air pollutant concentrations. In the model, the dependent variable is the concentration of the six pollutants at the national control point, and the independent variable is the original data monitored by the self-built point (– represents the variables eliminated in the model).

Independent variable	PM_2.5_	PM_10_	CO($\times {10}^{-2}$)	NO_2_	SO_2_	O_3_
Constant	8.663	47.475	2.127	174.759	− 303.100	63.734
PM_2.5_	0.724	0.890	0.005	0.070	–	–
PM_10_	–	–	–	–	0.034	− 0.032
CO	1.022	24.045	0.197	− 10.787	31.255	–
NO_2_	–	0.247	0.002	0.368	0.038	− 0.550
SO_2_	–	–	–	0.012	–	–
O_3_	–	–	–	− 0.148	0.081	0.264
Wind speed	–	–	− 0.033	− 14.472	− 2.268	12.520
Pressure	–	–	− 0.002	− 0.111	0.289	–
Precipitation	–	− 0.005	–	− 0.030	0.002	–
Temperature	–	–	–	–	–	2.188
Humidity	− 0.083	− 0.760	–	− 0.363	–	− 0.375
k value	0.050	0.040	0.010	0.020	0.020	0.050
F value	2307.828	1339.744	284.478	308.185	237.27	1123.756
P value	0.000	0.000	0.000	0.000	0.000	0.000
R^2^	0.860	0.781	0.431	0.451	0.388	0.750

### LASSO-NARX model construction

**Figure 5:**
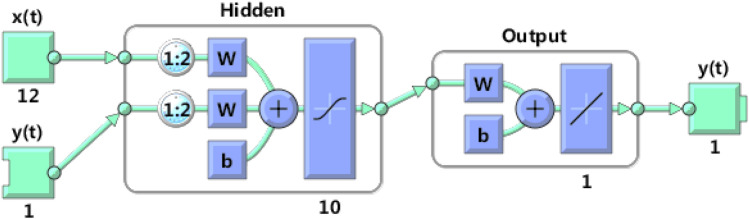
The frame structure of the LASSO-NARX model, where the input is the predicted value of the LASSO regression model and the measured value of the self-built point. This network has 12 inputs, 1 hidden layer with 10 hidden neurons, 2 input delay orders, and 1 linear output layer leading to 1 output.

The LASSO regression model gives a quantitative linear relationship between the pollutant concentration and various influencing factors^31^. However, there may be a nonlinear relationship between pollutant concentration and influencing factors, and the prediction accuracy of the LASSO model needs to be improved. Taking into account the time sequence of pollutant concentration, this paper uses NARX neural network to improve the accuracy of pollutant concentration prediction. We take the predicted value of LASSO regression and the data measured by self-built points as input, and the concentration of six pollutants as output to establish the NARX neural network model. The structure of the NARX neural network is shown in Fig. 5.

In the NARX neural network, it can be known from the Kolmogorov theorem that at most two hidden layers can identify arbitrary nonlinear characteristics, so this paper selects the default one hidden layer in Matlab. The number of nodes in the hidden layer of the neural network is determined by considering the training effect and training time. For the delay order in the model, determine the order change range based on experience, and find out the order when it no longer changes significantly as the model delay order according to the change of the mean square error of the model under different orders.

In the NARX model, the input is the predicted value of the LASSO regression model of O_3_, the concentration of six types of pollutants and five meteorological parameters measured by the self-built point, and the output is the O_3_ concentration measured by the national control point. 4135 samples are randomly divided into training set, validation set and test set at a ratio of 7:1.5:1.5. For comprehensive comparison, the input delay of NARX neural network is selected as 2, and the number of hidden layer nodes is 10. The training algorithm adopts the Levenberg–Marquardt algorithm with shorter training time, and the LASSO-NARX model is established with the help of Matlab software.

**Figure 6:**
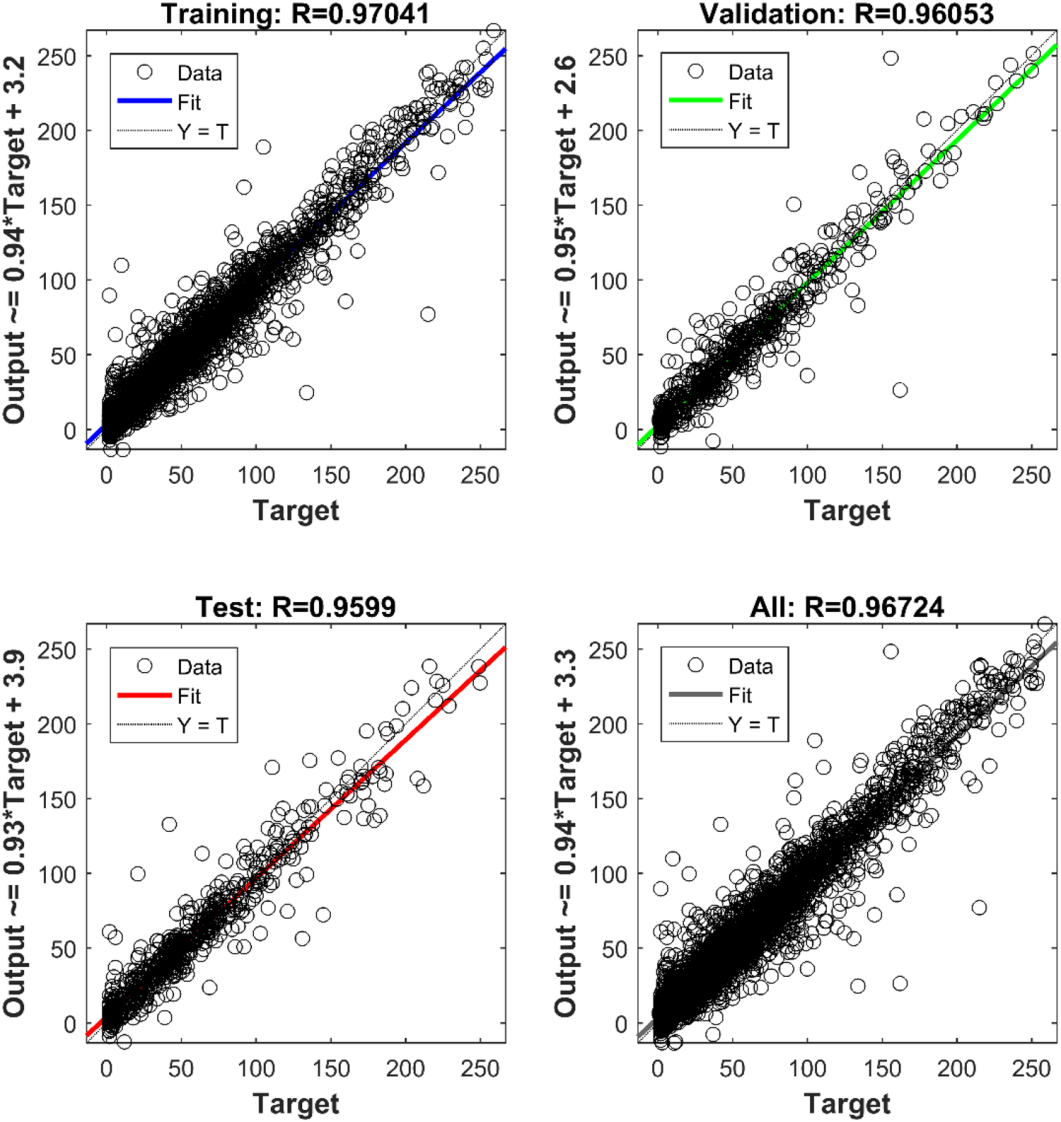
The prediction effect of O_3_’s LASSO-NARX model on the training set, validation set, test set and all sets.

**Figure 7:**
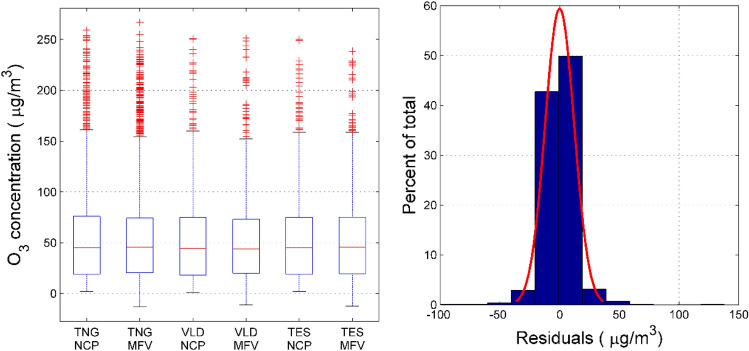
Residual test of LASSO-NARX model. Compare the national control point (NCP) measurement value and the model fit value (MFV) on the training set (TNG), validation set (VLD) and test set (TES) is seen on the left. The histogram of the residuals is seen on the right.

In order to visually show the prediction effect of the LASSO-NARX model, we have drawn the O_3_ concentration regression effect diagram. It can be seen from Fig. 6 that whether it is the training set, the validation set or the test set, the correlation coefficient between the predicted value of the model and the true value of the national control point exceeds 0.95, and the coefficients of each regression model are close to 1. It shows that the LASSO-NARX model has achieved good results in prediction. It can be seen from the box plot in Fig. 7 that regardless of the median, quantile, or outlier, the measured value of the national control point is roughly the same as the fitted value of the LASSO-NARX model. In addition, the boxplots of the training set, validation set and test set are also roughly the same. We conclude that the prediction and generalization ability of the LASSO-NARX model is good. It is worth noting that the output of the model is negative at several points where the concentration of O_3_ is particularly low at the national control point. In actual use, it can be considered that the O_3_ concentration is extremely low at this moment. It can be seen from the residual histogram that the error term roughly obeys the normal distribution, and the residual values are mostly distributed in [− 40, 40]. In this way, the LASSO-NARX model has been validated.

## Discussion

In the data calibration problem of the micro air quality detector, the LASSO model alone and the NARX neural network model alone can predict the concentration of pollutants. This paper also chooses a multilayer perceptron (MLP) and a radial basis function (RBF) neural network to compare with the LASSO-NARX model. Multilayer perceptron is a feedforward artificial neural network model that maps multiple input data sets to a single output data set. It introduces one or more hidden layers on the basis of a single-layer neural network, and the hidden layer is located between the input layer and the output layer. MLP is a neural network composed of fully connected layers, and the output of each hidden layer is transformed by an activation function. Radial basis function neural network is a type of forward network. It is based on the function approximation theory. It mainly contains input layer, radial base layer and output layer. Its hidden layer uses the radial basis function as the excitation function, which is an effective tool for identifying nonlinear systems^37,38^.

11 $$\sigma =\sqrt{\frac{1}{n}\sum_{i=1}^{n}{{(w}_{i}-\overline{w })}^{2}}$$

12 $${E}^{^{\prime}}=\sqrt{\frac{1}{n}\sum_{i=1}^{n}{{[(y}_{i}-\overline{y }){-(w}_{i}-\overline{w })]}^{2}}$$

**Figure 8:**
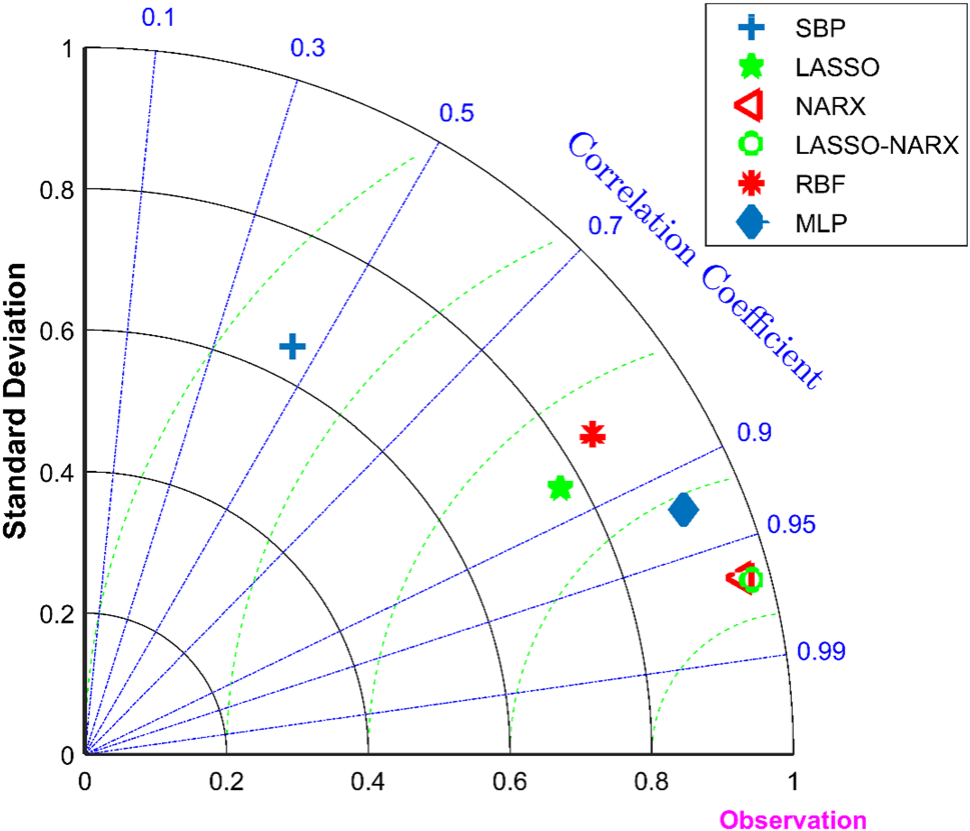
Taylor diagrams of predicted values of five models and measured values of self-built points, where SBP stands for self-built points.

Taylor diagrams are often used to visually compare the accuracy of various models^8^. The scattered points in the Taylor diagram represent the model, the radial line represents the correlation coefficient (Eq. (1)), the horizontal and vertical axis represents the standard deviation (Eq. (11)), and the dashed line represents the center root mean square error (Eq. (12)). Figure 8 is a Taylor analysis chart of O_3_ concentration. It should be noted that the indicators of each prediction model in the figure are based on the test set, but the self-built point (SBP) indicator is for the entire data set. It can be seen that compared with the O_3_ concentration measured by the national control point, the O_3_ concentration measured by the self-built point has the lowest accuracy, the LASSO model and the RBF neural network model have good accuracy, and the MLP neural network and NARX model have higher accuracy. The LASSO-NARX model proposed in this article performs best in comparison with other models.

Goodness of fit (R^2^), Root Mean Square Error (RMSE), Mean Absolute Error (MAE) and Relative Mean Absolute Percent Error (MAPE) can also be used to compare various air quality prediction models. Equation (10) and Eqs. (13)-(15) are specific formulas, where ${y}_{i}$ is the measured value at the national control point, $\overline{y }$ is the average value of the national control point, and ${w}_{i}$ is the regression value of the model^25,28^.

13 $$RMSE=\sqrt{\frac{1}{n}\sum_{i=1}^{n}{({y}_{i}-{w}_{i})}^{2}}$$

14 $$MAE=\frac{1}{n}\sum_{i=1}^{n}\left|{y}_{i}-{w}_{i}\right|$$

15 $$MAPE=\frac{1}{n}\sum_{i=1}^{n}\left|\frac{{y}_{i}-{w}_{i}}{{y}_{i}}\right|$$

It can be seen from Tables 4, 5, 6 and 7 that in the comparison with the data of the national air quality monitoring station, the measurement data of the micro air quality detector has a large error, so it needs to be calibrated. The LASSO regression model and RBF neural network model can calibrate self-built point data, but the effect needs to be improved. The MLP neural network and NARX model have a good effect on the calibration of self-built point data, and the LASSO-NARX model given in this article is the best in each evaluation index. In the index of goodness of fit, several self-built points are negative, which is caused by the large error of self-built points. Among the other three indexes, the most improved is the MAPE of NO_2_, which is an increase of 91.7%, and the least improved is the RMSE of PM_2.5_, which is an increase of 61.3%.

**Table 4:** R^2^ of six types of air pollutant concentrations between self-built points, model forecast values and national control point.

Input variable	Self-built points	LASSO	NARX	LASSO-NARX	RBF	SVR	MLP
PM_2.5_	0.551	0.860	0.931	0.933	0.667	0.933	0.907
PM_10_	− 1.076	0.781	0.909	0.918	0.558	0.938	0.827
CO	− 0.929	0.507	0.895	0.899	0.380	0.872	0.708
NO_2_	− 1.333	0.451	0.890	0.900	0.389	0.899	0.752
SO_2_	− 0.726	0.388	0.935	0.941	0.402	0.958	0.786
O_3_	0.094	0.750	0.932	0.936	0.715	0.945	0.864

**Table 5:** RMSE of six types of air pollutant concentrations between self-built points, model forecast values and national control point.

Input variable	Self-built points	LASSO	NARX	LASSO-NARX	RBF	SVR	MLP
PM_2.5_	22.436	12.515	8.800	8.687	19.323	8.649	10.777
PM_10_	66.263	21.495	13.911	13.208	30.570	11.656	19.126
CO	0.679	0.344	0.158	0.156	0.385	0.175	0.304
NO_2_	37.183	18.035	8.081	7.715	19.029	7.725	13.216
SO_2_	26.24	15.627	5.104	4.874	15.449	4.116	9.984
O_3_	45.673	24.003	12.477	12.190	25.638	11.304	18.603

**Table 6:** MAE of six types of air pollutant concentrations between self-built points, model forecast values and national control point.

Input variable	Self-built points	LASSO	NARX	LASSO-NARX	RBF	SVR	MLP
PM_2.5_	18.181	9.193	6.070	5.951	13.709	5.821	7.763
PM_10_	50.151	15.037	9.218	8.981	22.349	7.080	13.184
CO	0.549	0.263	0.100	0.098	0.288	0.110	0.237
NO_2_	29.838	13.877	4.924	4.806	14.166	4.658	9.991
SO_2_	12.867	10.421	2.684	2.464	9.998	2.116	7.246
O_3_	36.63	18.683	7.948	7.788	18.930	7.647	14.396

**Table 7:** MAPE of six types of air pollutant concentrations between self-built points, model forecast values and national control point.

Input variable	Self-built points	LASSO	NARX	LASSO-NARX	RBF	SVR	MLP
PM_2.5_	0.447	0.242	0.151	0.146	0.370	0.133	0.185
PM_10_	0.887	0.264	0.147	0.146	0.428	0.107	0.210
CO	0.478	0.317	0.096	0.095	0.379	0.112	0.283
NO_2_	2.129	0.760	0.1816	0.177	0.737	0.170	0.471
SO_2_	0.685	0.737	0.161	0.131	0.735	0.131	0.530
O_3_	4.322	1.487	0.428	0.397	1.446	0.373	1.002

## Conclusions

Low-cost micro air quality detectors can help humans conduct real-time and grid monitoring of the concentration of pollutants in the air. However, since the electrochemical sensor used by the micro air quality detector is susceptible to external influences, and after a period of use, it will exhibit range drift and zero point drift, so its measurement accuracy needs to be improved. The LASSO regression model can calibrate the data measured by the micro air quality detector and give the quantitative relationship between the pollutant concentration and each influencing factor, but it cannot find the nonlinear relationship between the pollutant concentration and each influencing factor. The NARX model can find the nonlinear relationship between the pollutant concentration and various influencing factors, and the prediction accuracy is significantly higher than the LASSO regression model. However, it cannot give a quantitative relationship between pollutant concentration and various influencing factors. The LASSO-NARX air quality combined model proposed in this study combines the advantages of the two models. It can not only reflect the quantitative relationship between the pollutant concentration and the influencing factors, but also has a higher prediction accuracy than the NARX neural network model alone. Using this model to calibrate the measurement data of the micro air quality detector can increase the accuracy by 61.3–91.7%. The LASSO-NARX model performs very well on the training set and test set, indicating that it has a strong generalization ability. The model uses a total of 4135 sets of data, and the data of the four seasons are all covered in the model, which also shows that the model is relatively stable. However, due to the different climatic conditions in different regions, this model may not be applicable to other regions. In the future, our team will try to collect data from other regions to further validate the model.
